# Yin Yang 1 is a target of microRNA-34 family and contributes to gastric carcinogenesis

**DOI:** 10.18632/oncotarget.2073

**Published:** 2014-06-07

**Authors:** An-Ming Wang, Tzu-Ting Huang, Kai-Wen Hsu, Kuo-Hung Huang, Wen-Liang Fang, Muh-Hwa Yang, Su-Shun Lo, Chin-Wen Chi, Jing-Jer Lin, Tien-Shun Yeh

**Affiliations:** ^1^ Institute of Biopharmaceutical Sciences, National Yang-Ming University, Taipei, Taiwan; ^2^ Department of Anatomy and Cell Biology, School of Medicine, National Yang-Ming University, Taipei, Taiwan; ^3^ Department and Institute of Pharmacology, School of Medicine, National Yang-Ming University, Taipei, Taiwan; ^4^ Department of Surgery, Taipei Veterans General Hospital, Taipei, Taiwan; ^5^ Institute of Clinical Medicine, School of Medicine, National Yang-Ming University, Taipei, Taiwan; ^6^ Department of Medicine, School of Medicine, National Yang-Ming University, Taipei, Taiwan; ^7^ Department of Surgery, National Yang-Ming University Hospital, Yi-Lan, Taiwan; ^8^ Department of Medical Research and Education, Taipei Veterans General Hospital, Taipei, Taiwan; ^9^ Institute of Biochemistry and Molecular Biology, National Taiwan University College of Medicine, Taipei, Taiwan; ^10^ Genome Research Center, National Yang-Ming University, Taipei, Taiwan; ^11^ Graduate Institute of Medical Sciences, College of Medicine, Taipei Medical University, Taipei, Taiwan

**Keywords:** YY1, miR-34 family, gastric cancer, carcinogenesis, pluripotency

## Abstract

Gastric cancer is the second leading cause of cancer-related death worldwide. Herein, we investigated the role of transcription factor Yin Yang 1 (YY1), a multi-functional protein, in tumorigenesis of gastric cancer cells. Results showed that YY1 contributed to gastric carcinogenesis of SC-M1 cells including growth, viability, and abilities of colony formation, migration, invasion, and tumorsphere formation. Levels of pluripotency genes CD44, Oct4, SOX-2, and Nanog were also up-regulated by YY1 in SC-M1 cells. Additionally, the 3'-untranslated region (3'-UTR) of YY1 mRNA was the target of microRNA-34 (miR-34) family consisting of miR-34a, miR-34b, and miR-34c. Overexpression of miR-34 family suppressed carcinogenesis through down-regulation of YY1 in NUGC-3 gastric cancer cells scarcely expressing miR-34 family. Alternatively, knockdown of miR-34 family promoted tumorigenesis *via* up-regulation of YY1 in SC-M1 and AZ521 gastric cancer cells with higher levels of miR-34 family. The miR-34 family also affected tumorsphere ultra-structure and inhibited the xenografted tumor growth as well as lung metastasis of SC-M1 cells through YY1. Expressions of miR-34a and miR-34c in gastric cancer tissues of patients were lower than those in normal tissues. Taken together, these results suggest that miR-34 family-YY1 axis plays an important role in the control of gastric carcinogenesis.

## INTRODUCTION

Gastric cancer is one of the most common cancers and the second leading cause of cancer-related death in the world [1]. Gastric cancer without distant metastasis is potentially curable by the surgical resection of its primary tumor and control of lymph node metastasis [2]. However, gastric cancer patients with distant metastasis still have poor prognosis at present. Both environmental and genetic factors contribute significantly to the risk of gastric tumorigenesis [3, 4]. So far, the mechanisms controlling the aggressiveness of gastric cancer have not yet been clearly characterized.

The ubiquitous transcription factor Yin Yang 1 (YY1) is a multi-functional protein acting as a repressor, activator, and initiator in transcriptional regulation [5, 6]. It plays an important role in the control of biological processes including proliferation, differentiation, apoptosis, and development [5-7]. YY1 is aberrantly expressed in several cancers [5-7] and also up-regulated in gastric cancer [8] as well as SIIA gastric cancer cells [9]. Recent studies showed that YY1 is implicated in carcinogenesis and can play either oncogenic or tumor-suppressive roles in tumor development and progression [5-7].

MicroRNAs (miRNAs) bind to the 3'-untranslated region (3'-UTR) of mRNAs and play a pivotal role in the regulation of many biological functions [10]. Increasing lines of evidence reveal that miRNAs could act either an oncogene or tumor suppressor in tumorigenesis [11-12]. Several studies reported the involvement of YY1 in modulating expression of miRNAs including miR-190 [13-14], miR-29 [15], and miR-206 [16]. Several reports also demonstrated that YY1 is the target of miR-34a [10, 17, 18], miR-7 [19], and miR-29 [15]. Furthermore, YY1 suppresses expression of miR-1 which in turn targets YY1 [20].

In the present study, we evaluated the role of YY1 in gastric carcinogenesis and also identified the tumor-suppressive miRNAs modulating gastric carcinogenesis through targeting YY1. There are the putative binding sites of miR-34 family consisting of miR-34a, miR-34b, and miR-34c. It had been reported that the restoration of miR-34a inhibits growth and tumorsphere formation in KATO III gastric cancer cells [21]. We further examined whether miR-34 family is involved in controlling tumor development and progression of gastric cancer cells *via* down-regulation of YY1 herein.

## RESULTS

### YY1 contributes to gastric carcinogenesis of SC-M1 cells

To assess whether any significant difference of YY1 mRNA expressions exists in stomach adenocarcinoma samples compared with those of normal tissues, data from The Cancer Genome Atlas (TCGA) were analyzed. Results showed that levels of YY1 mRNA were significantly increased in numerous stomach adenocarcinoma samples compared with normal tissue samples (Supplementary Figure S1, *left*). Furthermore, expressions of YY1 mRNA were higher in stomach adenocarcinoma samples than in their corresponding normal-tissue counterparts from 30 patients with stomach adenocarcinoma (Supplementary Figure S1, *right*).

**Figure 1:**
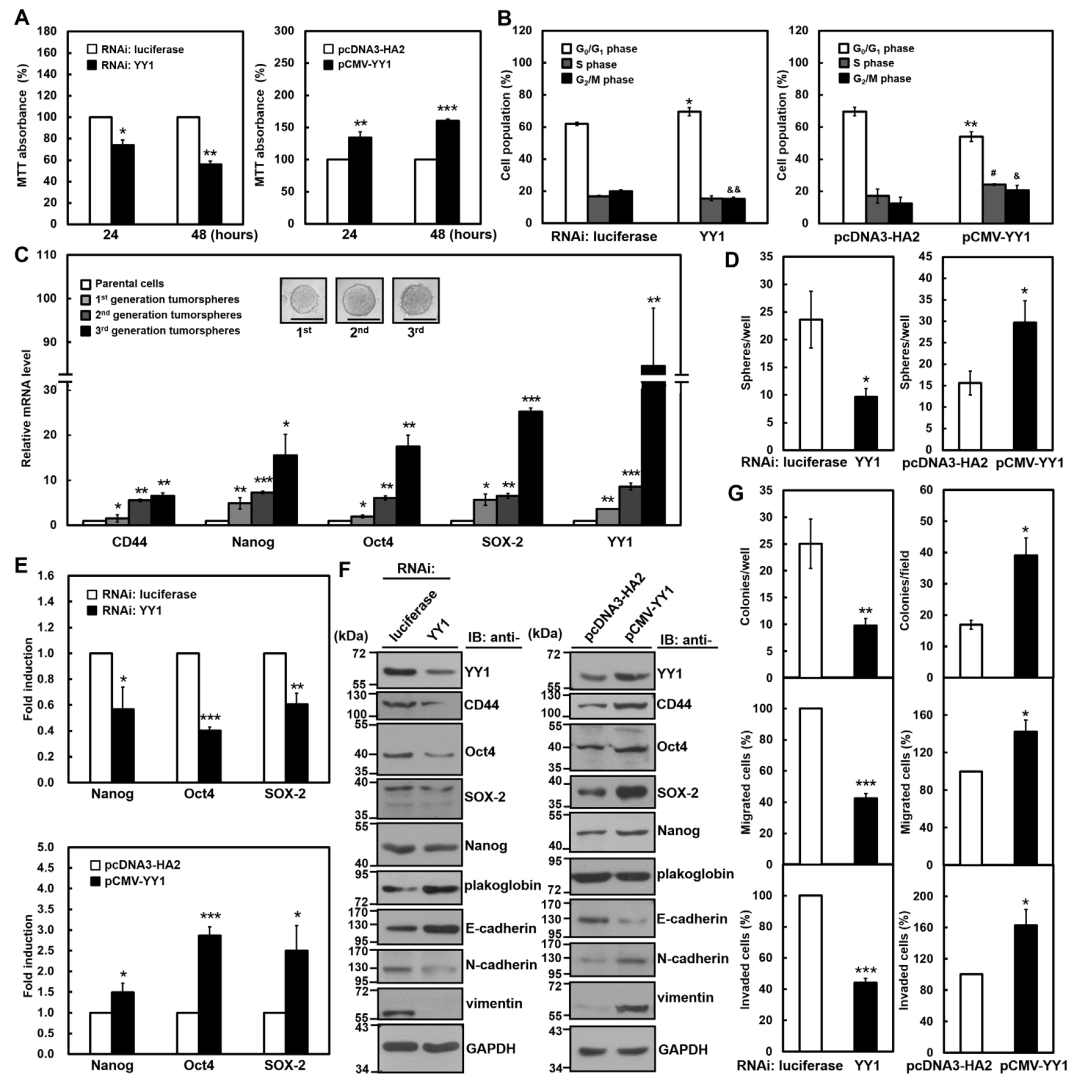
YY1 contributes to gastric carcinogenesis in SC-M1 cells SC-M1 cells were transfected with siRNA vectors against YY1 or luciferase and YY1-expressing construct pCMV-YY1 or control vector pcDNA3-HA2 for 48 hours. (A) The transfected cells were seeded onto 24-well plates and then incubated for 24 or 48 hours to analyze cell viability by MTT assay. *, *P* < 0.05; **, *P* < 0.01; ***, *P* < 0.001 compared with cells transfected with siRNA vector against luciferase or control vector pcDNA3-HA2. (B) The transfected cells were stained with PI to analyze their DNA contents by flow cytometry. Cell proportions in G_0_/G_1_, S, and G_2_/M phases of cell cycle were assayed. *, *P* < 0.05; **, *P* < 0.01; #, *P* < 0.05; &, *P* < 0.05; &&, *P* < 0.01 compared with cells transfected with siRNA vector against luciferase or control vector pcDNA3-HA2 in G_0_/G_1_, S, and G_2_/M phases, respectively. (C) A total of 500 or 1,000 SC-M1 cells were seeded onto 24-well ultra-low attachment plates under stem cell-selective conditions for the subsequent formation assay of the first, second, and third generation tumorspheres. The transcript levels of CD44, Nanog, Oct4, SOX-2, and YY1 were measured by quantitative real-time PCR and then normalized to GAPDH. *, *P* < 0.05; **, *P* < 0.01; ***, *P* < 0.001 compared with parental cells. The upper islets are representative images of tumorspheres. Bar, 100 μm. (D) The transfected cells were seeded and then incubated for 9 days for tumorsphere formation assay. *, *P* < 0.05 compared with cells transfected with siRNA vector against luciferase or control vector pcDNA3-HA2. (E) After co-transfection with siRNA vector against YY1 (*upper*) or YY1-expressing construct pCMV-YY1 (*lower*) and reporter plasmids Nanog-Luc (Nanog), Oct4-Luc (Oct4), or SOX-2-Luc (SOX-2) for 48 hours, SC-M1 cells were harvested for reporter gene assay. *, *P* < 0.05; **, *P* < 0.01; ***, *P* < 0.001 compared with cells transfected with siRNA vector against luciferase or control vector. (F) Whole-cell extracts were prepared from SC-M1 cells transfected with siRNA vectors against YY1 or luciferase (*left*) and YY1-expressing construct pCMV-YY1 or control vector pcDNA3-HA2 (*right*). Western blot analysis was performed using anti-YY1, anti-CD44, anti-Oct4, anti-SOX-2, anti-Nanog, anti-plakoglobin, anti-E-cadherin, anti-N-cadherin, anti-vimentin, and anti-GAPDH antibodies. (G) SC-M1 cells transfected with siRNA vectors against YY1 or luciferase (*left*) and YY1-expressing construct pCMV-YY1 or control vector pcDNA3-HA2 (*right*) were seeded for colony formation (*upper*), migration (*middle*), and invasion (*lower*) assays. *, *P* < 0.05; **, *P* < 0.01; ***, *P* < 0.001 compared with cells transfected with siRNA vector against luciferase or control vector. Data are representative of the mean values and standard deviations from at least 3 independent experiments.

To evaluate the role of YY1 in gastric tumorigenesis, we first sought to check whether YY1 modulates the growth of gastric cancer cells. Because more than 95% of malignancies of stomach are adenocarcinomas, human stomach adenocarcinoma SC-M1 cells were used in the present study. The 3-(4,5-dimethyl-2-thiazolyl)-2,5-diphenyl tetrazolium bromide (MTT) assay and propidium iodide (PI)-staining in combination with flow cytometry analysis were performed after transfection with the small interfering RNA (siRNA) vector against YY1 for knockdown or with the expression construct of YY1 for overexpression. Results of MTT assay showed that the viability of SC-M1 cells was suppressed by YY1 knockdown but enhanced by YY1 overexpression (Figure 1A). Data of flow cytometry analysis showed that cells were slightly increased in G_0_/G_1_ phase by YY1 knockdown (Figure 1B). Whereas the cell population in G_0_/G_1_ phase was decreased along with the increment of those in S and G_2_/M phases by YY1 overexpression.

Subsequently, it was further addressed whether YY1 is involved in the maintenance of cancer stem-like phenotype in gastric cancer cells by examining the ability of tumorsphere formation. The tumorspheres of first generation in SC-M1 cells were found after incubation for 6 days under non-adherent condition with stem cell-selective medium (Figure 1C). Using quantitative real-time PCR analysis, mRNA levels of pluripotency genes were enhanced in SC-M1 cells under stem cell-selective conditions including CD44, Nanog, Oct4, and SOX-2 compared with those of parental cells. Notably, YY1 mRNA expression was also elevated in the first-generation tumorspheres of SC-M1 cells. Similar results were obtained in the second- and third-generation tumorspheres of SC-M1 cells (Figure 1C). Interestingly, the higher generation of tumorspheres exhibited the more levels of CD44, Nanog, Oct4, SOX-2, and YY1 mRNAs.

Ability of tumorsphere formation in SC-M1 cells was repressed by YY1 knockdown, whereas promoted by YY1 overexpression (Figure 1D). The activities of reporter genes containing promoters of pluripotency genes were inhibited by YY1 knockdown in SC-M1 cells including Nanog, Oct4, and SOX-2, whereas elevated by YY1 overexpression (Figure 1E). CD44, Oct4, SOX-2, and Nanog levels were decreased by YY1 knockdown in SC-M1 cells, but increased by YY1 overexpression (Figure 1F).

Moreover, levels of epithelial markers plakoglobin and E-cadherin were enhanced by YY1 knockdown in SC-M1 cells, whereas expressions of mesenchymal markers N-cadherin and vimentin were decreased (Figure 1F, *left*). In reciprocal, levels of plakoglobin and E-cadherin were down-regulated by YY1 overexpression in SC-M1 cells along with the up-regulated expressions of N-cadherin and vimentin (Figure 1F, *right*). To investigate whether YY1 participates in gastric cancer progression, abilities of colony formation, migration, and invasion were examined. These abilities in SC-M1 cells were attenuated by YY1 knockdown, whereas increased by YY1 overexpression (Figure 1G).

### YY1 is the target gene of miR-34 family members

**Figure 2:**
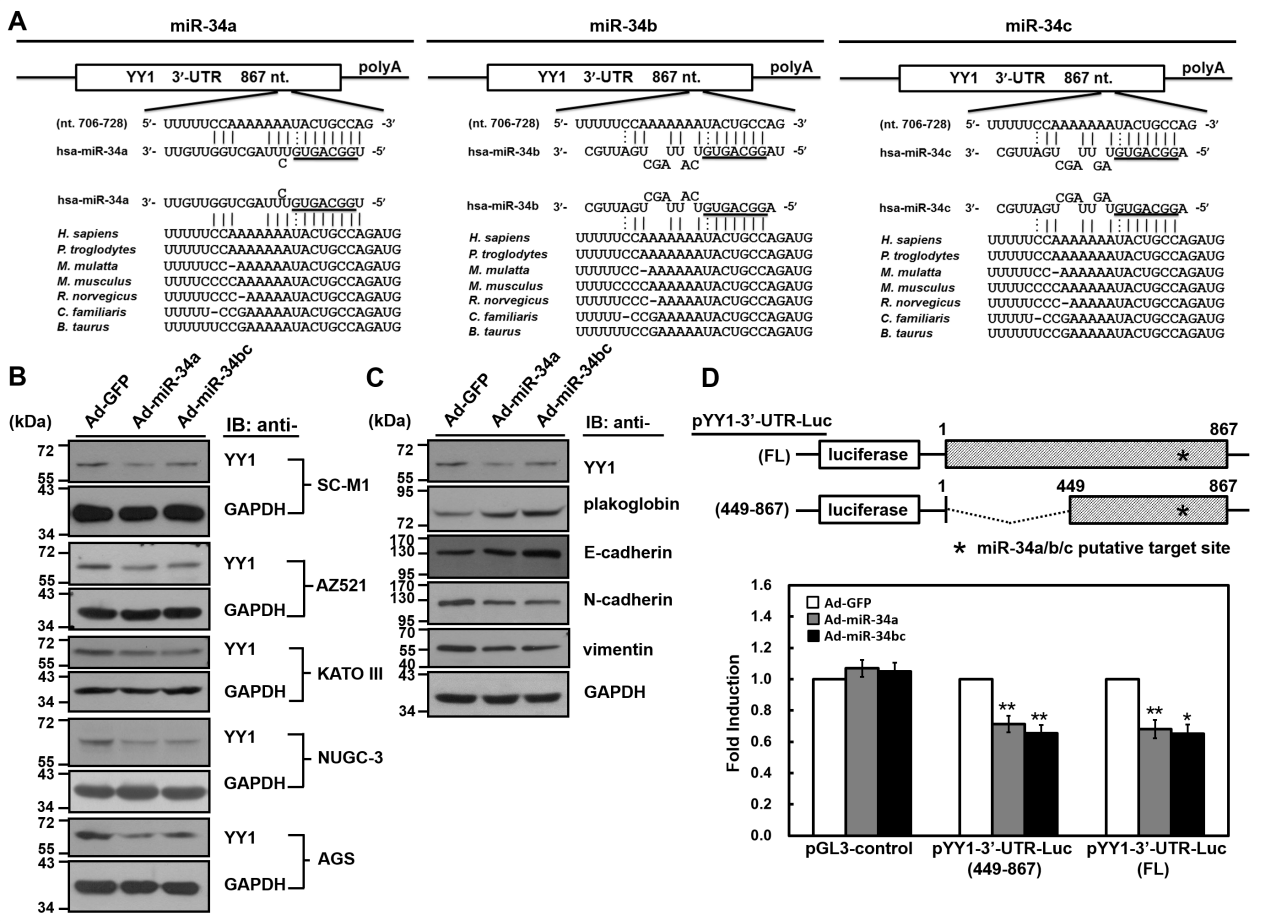
YY1 is the target gene of miR-34 family members (A) There is a putative miR-34 family-binding site located at nucleotide 720 to 726 from the start of 3'-UTR of human YY1 mRNA as predicted by TargetScan 5.2, PicTar, and miRecords algorithms. The sequences of miR-34 family are aligned with the 3'-UTRs of YY1 in human (*H. sapiens*), chimpanzee (*P. troglodytes*), monkey (*M. mulatta*), mouse (*M. musculus*), rat (*R. norvegicus*), dog (*C. familiaris*), and cow (*B. taurus*). (B) SC-M1, AZ521, KATO III, NUGC-3, and AGS cells were infected with adenoviruses expressing miR-34a (Ad-miR-34a), miR-34b and miR-34c (Ad-miR-34bc), or GFP (Ad-GFP) for 48 hours. Whole-cell extracts of the infected cells were prepared for Western blot analysis using anti-YY1 and anti-GAPDH antibodies. (C) Whole-cell extracts of SC-M1 cells infected with adenoviruses expressing miR-34a, miR-34b and miR-34c, or GFP were prepared for Western blot analysis using anti-YY1, anti-plakoglobin, anti-E-cadherin, anti-N-cadherin, anti-vimentin, and anti-GAPDH antibodies. (D) Schematic representation of luciferase reporter plasmids pYY1 3'-UTR-Luc (FL) and (449-867) containing DNA fragments of full-length (nucleotide 1-867) and truncated (nucleotide 449 to 867) human YY1 3'-UTRs, respectively (*upper*). Star indicates position of the putative miR-34 family-binding site in reporter plasmids. After transfection with reporter plasmids pYY1 3'-UTR-Luc (FL) and (449-867) for 24 hours, SC-M1 cells were infected with adenoviruses expressing miR-34a, miR-34b and miR-34c, or GFP for 24 hours for reporter gene assay (*lower*). Means of three independent experiments performed at least in triplicate are shown. *, *P* < 0.05; **, *P* < 0.01 compared with cells infected with adenoviruses expressing GFP.

To identify tumor-suppressive miRNAs modulating gastric carcinogenesis through targeting YY1, we employed widely used software including TargetScan 5.2, PicTar, and miRecords algorithms to search for the putative binding sites of miRNAs in the 3'-UTR of human YY1 mRNA. Our *in silico* analyses showed that the putative binding sites of miR-34a, miR-34b, and miR-34c reside at nucleotide 720 to 726 from the start of YY1 3'-UTR (Figure 2A). There is the phylogenic conservation of the putative miR-34a, miR-34b, and miR-34c-binding sites within 3'-UTRs of YY1 mRNAs in mammalian species. Therefore, members of miR-34 family could be potential regulators of YY1 expression.

To further evaluate whether YY1 is a target of miR-34 family, the adenoviral system exogenously expressing miR-34 family was established. Owing to miR-34b and miR-34c (referred to hereafter as miR-34bc) are located within the same primary transcript [22], it was established to simultaneously express them in the adenoviral system. As revealed by miRNA quantitative real-time PCR analysis (Supplementary Figure S2), levels of miR-34 family members were increased in SC-M1 and AZ521 gastric cancer cells infected with miR-34a- or miR-34bc-expressing adenoviruses as compared with those infected with green fluorescent protein (GFP)-expressing adenoviruses. YY1 expressions were decreased after infection with miR-34a- or miR-34bc-expressing adenoviruses by Western blot analysis in SC-M1, AZ521, KATO III, NUGC-3, and AGS gastric cancer cells (Figure 2B). In addition, levels of epithelial markers plakoglobin and E-cadherin were enhanced along with the decreased expression of mesenchymal markers N-cadherin and vimentin in SC-M1 cells after infection with miR-34a- or miR-34bc-expressing adenoviruses (Figure 2C).

Next, luciferase reporter gene assay was performed to check whether miR-34 family targets YY1 3'-UTR. DNA fragments containing full-length or truncated YY1 3'-UTRs were inserted at the rear of luciferase reporter gene to construct pYY1 3'-UTR-Luc reporter plasmids (Figure 2D). After transfection with these reporter plasmids, reporter gene activities were inhibited in SC-M1 cells infected with miR-34a- or miR-34bc-expressing adenoviruses as compared to those infected with GFP-expressing adenoviruses.

### Negative correlation between YY1 and miR-34b or miR-34c levels in gastric cancer cells

**Figure 3:**
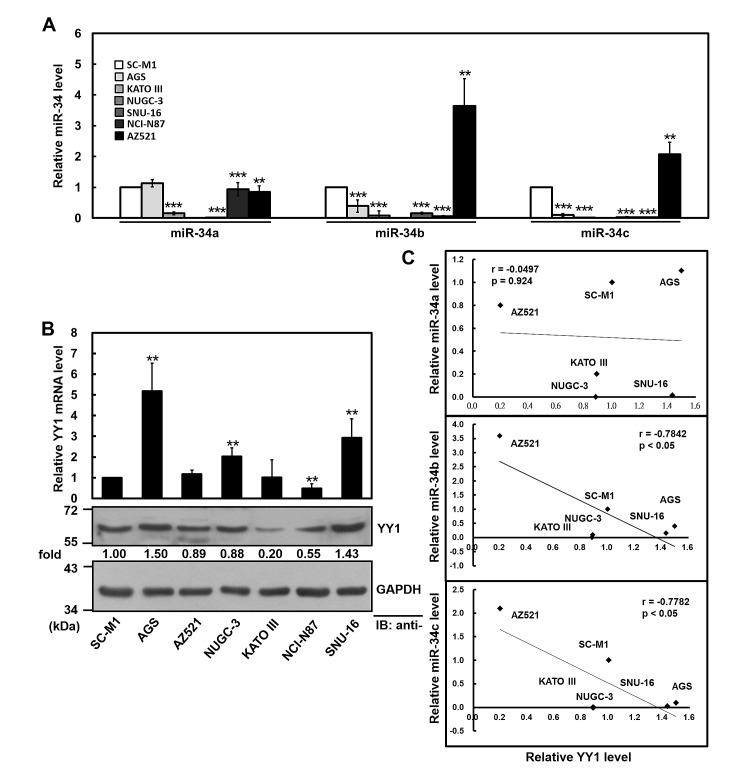
Negative correlation between YY1 and miR-34 family levels in gastric cancer cells (A) The relative levels of endogenous miR-34 family in gastric cancer cells were determined using miRNA quantitative real-time PCR including AZ521, AGS, KATO III, NUGC-3, SNU-16, NCI-N87, and SC-M1 cells. The level of miR-34 family in SC-M1 cells was set to unity. **, *P* < 0.01; ***, *P* < 0.001 compared with SC-M1 cells. (B) The transcript levels of YY1 in gastric cancer cells were measured by quantitative real-time PCR (*upper*). The data were compared, after being normalized to GAPDH. The level of YY1 mRNA in SC-M1 cells was set to unity. **, *P* < 0.01 compared with SC-M1 cells. Whole-cell extracts of these cells were also prepared for Western blot analysis using anti-YY1 and anti-GAPDH antibodies (*lower*). Their intensities were quantified by Multi-Gouge V3.0 and normalized to the internal control GAPDH. The results were calibrated to YY1 level in SC-M1 cells. (C) The correlation between miR-34 family and YY1 protein expressions in six gastric cancer cells was analyzed by Pearson correlation analysis.

Both miRNA quantitative real-time PCR and Western blot analysis were employed to explore the relationship between endogenous YY1 and miR-34 family levels in gastric cancer cells including AZ521, AGS, KATO III, NUGC-3, SNU-16, NCI-N87, and SC-M1 cells. Among these cells, levels of miR-34 family were higher in AZ521 and SC-M1 cells, while hardly detected in KATO III, NUGC-3 and SNU-16 cells (Figure 3A). Although there were differential levels of YY1 mRNA and protein in these cells, they were abundantly expressed (Figure 3B). AGS and SNU-16 cells exerted the higher levels of YY1 protein among these cells, whereas KATO III and NCI-N87 cells showed the lower levels. Using the Pearson correlation analysis, we found that the relative levels of miR-34b and miR-34c but not miR-34a were inversely proportional to YY1 protein expression in these cells excluding NCI-N87 cells (Figure 3C and Supplementary Figure S3).

### Exogenous YY1 expression restores miR-34 family-suppressed gastric carcinogenesis

**Figure 4:**
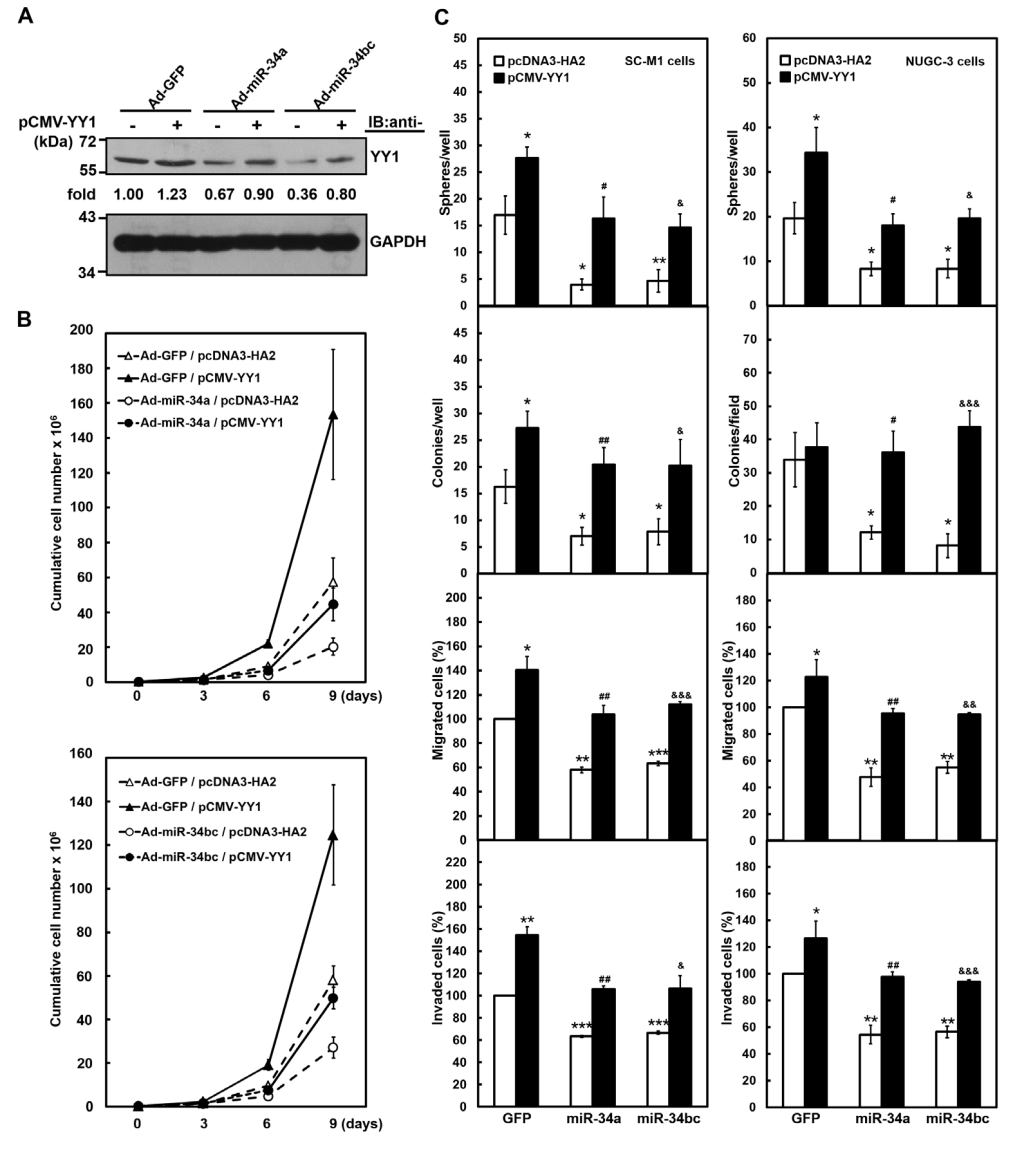
Exogenous YY1 restores gastric carcinogenesis suppressed by miR-34 family After transfection with YY1-expressing construct pCMV-YY1 lacking 3'-UTR sequence (+) or control vector (-) for 24 hours, SC-M1 or NUGC-3 cells were infected with adenoviruses expressing miR-34a (Ad-miR-34a), miR-34b and miR-34c (Ad-miR-34bc), or GFP (Ad-GFP) for another 24 hours. (A) Whole-cell extracts of the treated SC-M1 cells were prepared for Western blot analysis using anti-YY1 and anti-GAPDH antibodies. Their intensities were quantified and then normalized to the internal control GAPDH as described in the legend to Figure 3. (B) After transfection with YY1-expressing construct pCMV-YY1 or control vector pcDNA3-HA2 and subsequent infection with adenoviruses expressing miR-34a (*upper*), miR-34b and miR-34c (*lower*), or GFP, SC-M1 cells (3 × 10^5^) were seeded and then counted by trypan blue exclusion method at the time indicated. (C) The treated SC-M1 (*left*) or NUGC-3 (*right*) cells were seeded for the subsequent assays of tumorsphere formation, colony formation, migration, and invasion assays as described in the legend to Figure 1. *, *P* < 0.05; **, *P* < 0.01; ***, *P* < 0.001 compared with cells transfected with mock and infected with adenoviruses expressing GFP. #, *P* < 0.05; ##, *P* < 0.01 compared to cells transfected with mock and infected with adenoviruses expressing miR-34a. &, *P* < 0.05; &&, *P* < 0.01; &&&, *P* < 0.001 compared to cells transfected with mock and infected with adenoviruses expressing miR-34b as well as miR-34c.

We also sought to unravel whether tumor suppressor miR-34 family inhibits gastric carcinogenesis *via* down-regulating YY1 expression. Data of Western blot analysis showed that YY1 levels down-regulated by infection with miR-34a- or miR-34bc-expressing adenoviruses were restored after transfection with miR-34 family-insensitive YY1-expressing construct in SC-M1 cells (Figure 4A). Results of trypan blue exclusion method showed that growth of SC-M1 cells was inhibited after infection with miR-34a- or miR-34bc-expressing adenoviruses (Figure 4B). The inhibition of growth by miR-34a or miR-34bc was rescued by YY1 overexpression in SC-M1 cells.

Furthermore, the suppressive effect of miR-34 family on the abilities of tumorsphere formation, colony formation, migration, and invasion in SC-M1 cells was restored by YY1 overexpression (Figure 4C, *left*). Similarly, the exogenous YY1 expression also relieved the miR-34 family-mediated reduction of tumorsphere formation, colony formation, migration, and invasion abilities in NUGC-3 cells that scarcely expressed miR-34 family (Figure 4C, *right*). NUGC-3 cells were further used to evaluate the effect of miR-34 family on morphological change of gastric cancer cells. Parental and GFP-expressing adenoviruses-infected NUGC-3 cells dispersedly grew and had a little spindle- and fibroblast-like morphology, whereas those cells infected with miR-34a- or miR-34bc-expressing adenoviruses grew as clusters of cells (Supplementary Figure S4). YY1 overexpression induced morphological change in NUGC-3 cells infected with miR-34 family-expressing adenoviruses from the tightly packed colonies to more extended and elongated shape.

### YY1 knockdown attenuates antagomir-34-promoted gastric carcinogenesis

**Figure 5:**
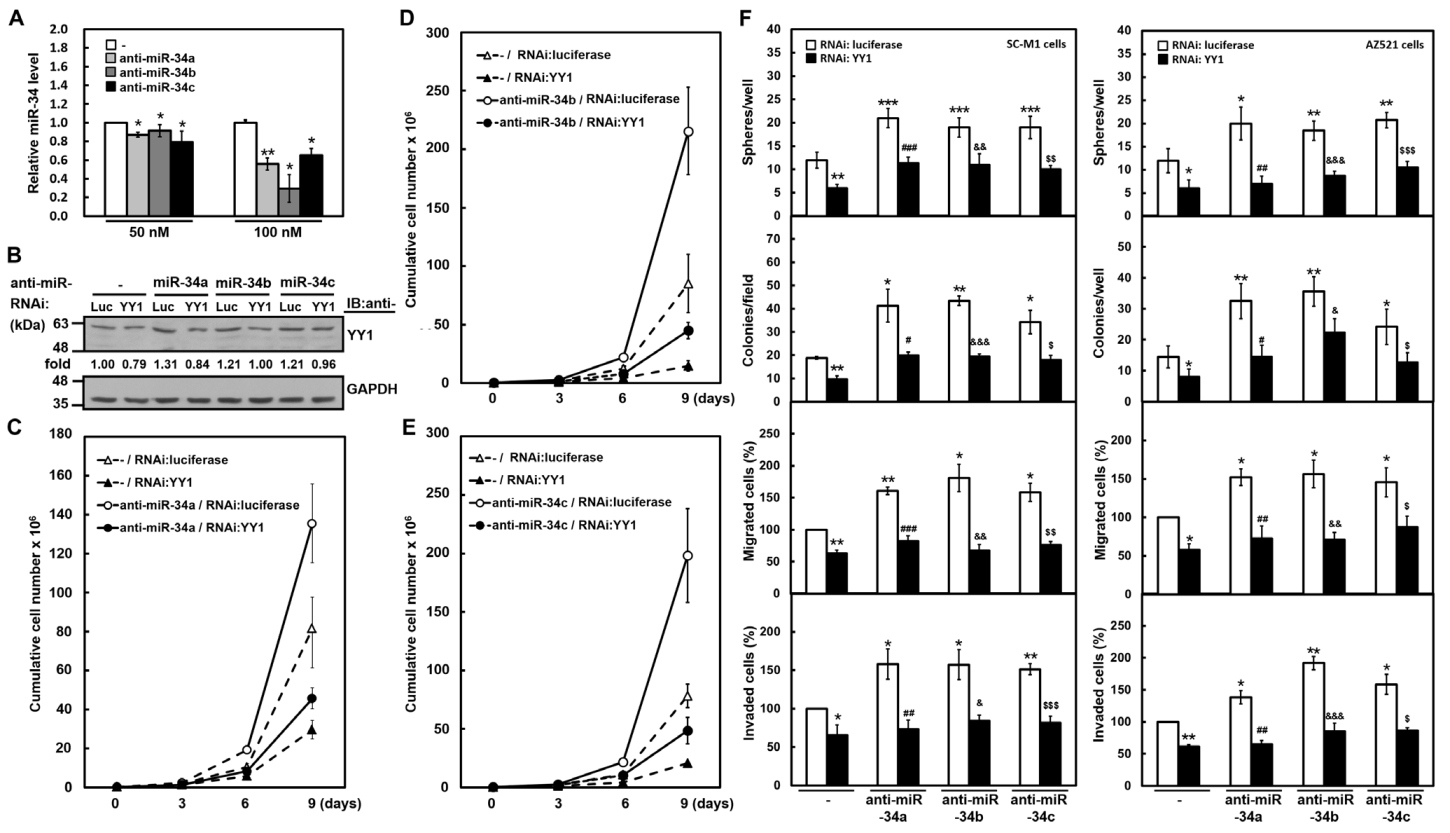
YY1 knockdown attenuates gastric carcinogenesis promoted by antagomir-34 treatment (A) SC-M1 cells were transfected with 50 or 100 nM antogomir-34a (anti-miR-34a), antogomir-34b (anti-miR-34b), antogomir-34c (anti-miR-34c) or scrambled control (-) for 48 hours. The transcript levels of miR-34 family in the transfected cells were measured by miRNA quantitative real-time PCR. *, *P* < 0.05; **, *P* < 0.01 compared with cells transfected with 50 or 100 nM scrambled controls. (B-E) One hundred nM antagomir-34a, antagomir-34b, antagomir-34c, or scrambled control (-) were co-transfected with siRNA vectors against YY1 or luciferase (Luc) into SC-M1 cells for 48 hours. The YY1 levels of the transfected SC-M1 cells were detected by Western blot analysis (B). As described in the legend to Figure 3, their intensities were quantified and then normalized to the internal control GAPDH. Cells co-transfected with siRNA vectors against YY1 or luciferase and antagomir-34a (C), antagomir-34b (D), or antagomir-34c (E) were also seeded and then counted by trypan blue exclusion method at the time indicated as described in the legend to Figure 1. (F) SC-M1 (*left*) or AZ521 (*right*) cells were co-transfected with antogomir-34a, antogomir-34b, or antogomir-34c and siRNA vector against YY1 for 48 hours. Then the transfected cells were seeded for the assays of tumorsphere formation, colony formation, migration, and invasion as described in the legend to Figure 1. *, *P* < 0.05; **, *P* < 0.01; ***, *P* < 0.001 compared with cells co-transfected with siRNA vector against luciferase and scrambled control. #, *P* < 0.05; ##, *P* < 0.01; ###, *P* < 0.001 compared to cells co-transfected with siRNA vector against luciferase and antogomir-34a. &, *P* < 0.05; &&, *P* < 0.01; &&&, *P* < 0.001 compared to cells co-transfected with siRNA vector against luciferase and antogomir-34b. $, *P* < 0.05; $$, *P* < 0.01; $$$, *P* < 0.001 compared to cells co-transfected with siRNA vector against luciferase and antogomir-34c.

Alternatively, we also addressed the role of endogenous miR-34 family-YY1 axis in gastric carcinogenesis by knockdown. Antagomir-34a, -34b, and -34c reagents, chemically modified antisense RNA oligonucleotides, were employed to inhibit the function of endogenous miR-34 family. Using analysis of miRNA quantitative real-time PCR, transient transfection with 50 or 100 nM of antagomir-34a, -34b, and -34c significantly knocked down miR-34a, miR-34b, and miR-34c expressions in SC-M1 cells, respectively (Figure 5A). The YY1 levels of SC-M1 cells were enhanced after knockdown of miR-34 family using Western blot analysis (Figure 5B). This enhancement of YY1 expression was attenuated by YY1 knockdown. Growth of SC-M1 cells was promoted by miR-34 family knockdown as determined by trypan blue exclusion method (Figure 5C-5E). The promotion of growth by miR-34 family knockdown was abolished after YY1 knockdown in SC-M1 cells. Additionally, the augmented effect on the abilities of tumorsphere formation, colony formation, migration, and invasion in SC-M1 cells by miR-34 family knockdown was suppressed after YY1 knockdown (Figure 5F, *left*). Likewise, the increased effect on gastric carcinogenesis by miR-34 family knockdown was abrogated in AZ521 cells with higher level of miR-34 family after YY1 knockdown (Figure 5F, *right*).

### miR-34 family affects tumorsphere ultra-structure of SC-M1 cells through YY1

**Figure 6:**
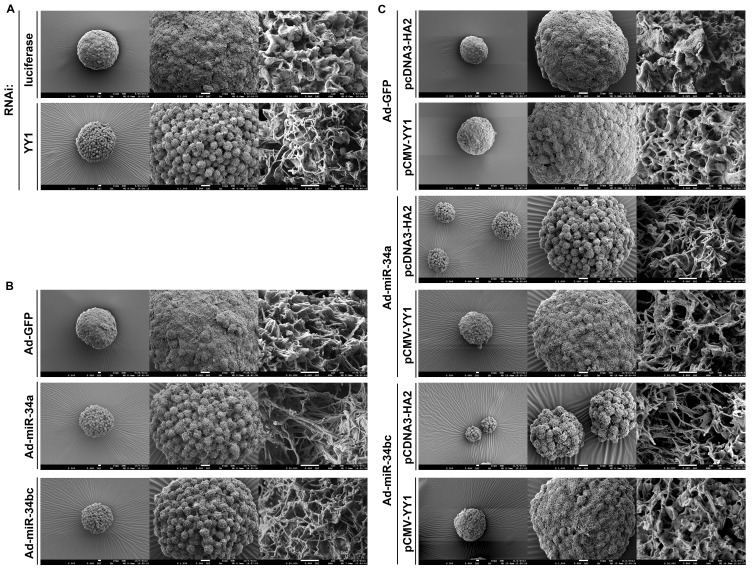
miR-34 family affects tumorsphere ultra-structure of SC-M1 gastric cancer cells through YY1 (A-B) SC-M1 cells were transfected with siRNA vector against YY1 or luciferase (A) and infected with adenoviruses expressing miR-34a, miR-34b and miR-34c, or GFP (B) for the subsequent assay of tumorsphere formation as described in the legend to Figure 1. (C) After transfection with YY1-expressing construct pCMV-YY1 or control vector pcDNA3-HA2 and subsequent infection with adenoviruses expressing miR-34a, miR-34b and miR-34c, or GFP, SC-M1 cells were seeded for the subsequent formation assay of tumorspheres. The ultra-structure of tumorspheres was visualized by scanning electron microscope. Images at 300×(*left*), 1,000×(*middle*), and 20,000×(*right*) magnification are from a representative experiment. Scale bar: *left*, 10 μm; *middle*, 10 μm; *right*, 1 μm.

The effect of YY1 and miR-34 family on the ultra-structure of tumorspheres in gastric cancer cells was also examined by scanning electron microscope. Results showed that tumorsphere surface of SC-M1 cells was a microvilli-like structure (Figure 6A), which had also been observed in neuroblastoma tumorspheres [23]. YY1 knockdown reduced the microvillus extension and caused the change of morphology in tumorsphere ultra-structure of SC-M1 cells. Likewise, the ectopic miR-34a or miR-34bc diminished the microvillus extension and affected the tumorsphere ultra-structure in SC-M1 cells (Figure 6B). The reduction of microvillus extension and morphological change in tumorspheres by miR-34a or miR-34bc overexpressions were rescued by YY1 overexpression in SC-M1 cells (Figure 6C).

### miR-34 family regulates tumor growth and lung metastasis of SC-M1 gastric cancer cells through YY1

**Figure 7:**
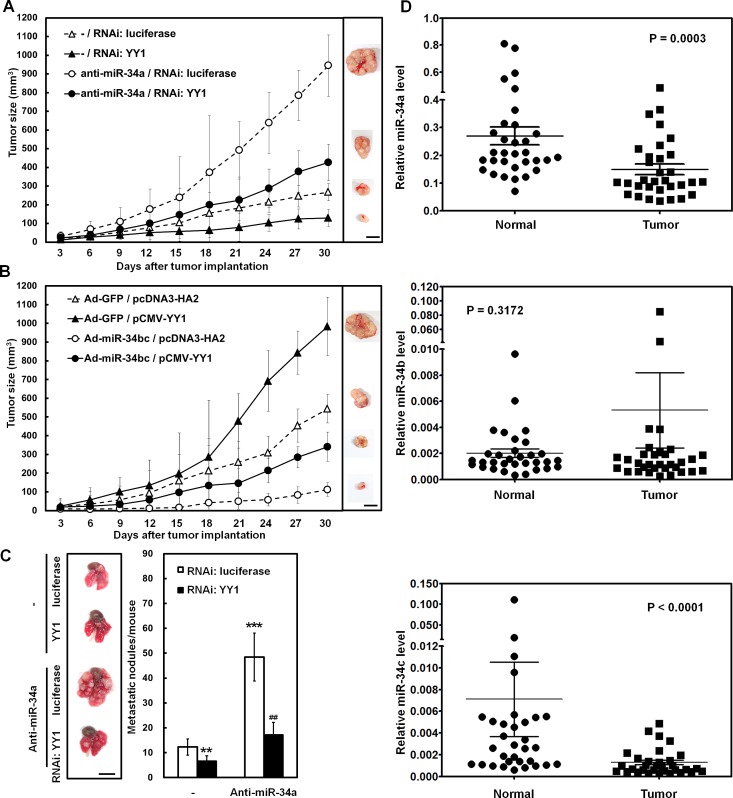
miR-34 family inhibits tumor growth and lung metastasis of SC-M1 gastric cancer cells through down-regulation of YY1 (A) After co-transfection with antogomir-34a (anti-miR-34a) or scrambled control (-) and siRNA vectors against YY1 or luciferase into SC-M1 cells for 48 hours, the viable transfected cells were subcutaneously injected into nude mice (n= 5 per group) for the measurement of tumor sizes at the time indicated. (B) After transfection with YY1-expressing construct pCMV-YY1 or control vector pcDNA3-HA2 for 24 hours, the transfected cells were infected with adenoviruses expressing miR-34b and miR-34c (Ad-miR-34bc) or GFP (Ad-GFP) for another 24 hours. The viable infected cells were subcutaneously injected into nude mice (n= 5 per group) for the measurement of tumor sizes. (C) After co-transfection with antogomir-34a or scrambled control (-) and siRNA vectors against YY1 or luciferase into SC-M1 cells for 48 hours, the viable transfected cells were injected into NOD-SCID mice (n= 7 per group) by tail vein injection for measurement of metastatic nodules in lungs. After 16 weeks, the mice were sacrificed and the metastatic nodules in the lungs were counted by gross and microscopic examination. **, *P* < 0.01; ***, *P* < 0.001 compared with cells co-transfected with scrambled control (-) and siRNA vector against luciferase. ##, *P* < 0.01 compared to cells co-transfected with antogomir-34a and siRNA vector against luciferase. Data are from a representative experiment that was performed two times with similar results. Bar, 1.0 cm. (D) Tumor and the adjacent non-tumor tissue pairs from 32 gastric cancer patients were examined using miRNA quantitative real-time PCR analysis. Levels of miR-34a, miR-34b, and miR-34c in the gastric cancer tissues were compared with those of the corresponding adjacent normal tissues.

The xenografted tumor growth in the case of subcutaneous injection with SC-M1 cells in nude mice was also checked to assess the function of miR-34 family-YY1 axis in tumor growth of gastric cancer cells *in vivo*. As demonstrated by Suzuki *et al*. [24], miR-34a is abundantly expressed in gastric cancer cells, whereas miR-34b and miR-34c are epigenetically silenced. Therefore, we knocked down miR-34a or over-expressed miR-34b as well as miR-34c in SC-M1 cells to perform tumor growth assay in the present study. Results showed that the tumor sizes of YY1-knocked down SC-M1 cells were smaller than those of control cells (Figure 7A). Knockdown of miR-34a promoted the tumor sizes of SC-M1 cells. The increment of tumor sizes in SC-M1 cells by miR-34a knockdown was relieved after YY1 knockdown. On day 30 after implantation, the mice were sacrificed and then subcutaneous tumors were excised for the detection of YY1 mRNA and miR-34a expressions. As determined by quantitative real-time PCR, the levels of YY1 mRNA in the xenografted tumors of YY1-knocked down SC-M1 cells were down-regulated compared to those of control cells (Supplementary Figure S5, *left*). Results of miRNA quantitative real-time PCR showed that miR-34a levels of the xenografted tumors in mice injected with miR-34a-knocked down SC-M1 cells were lower than those of control cells (Supplementary Figure S5, *right*).

The xenografted tumor sizes of SC-M1 cells transfected with YY1-expressing construct were larger than those of control cells (Figure 7B). Infection with miR-34bc-expressing adenoviruses suppressed the tumor sizes of SC-M1 cells as compared with those infected with GFP-expressing adenoviruses. The miR-34bc-inhibited tumor sizes of SC-M1 cells were restored by YY1 overexpression. On day 30 postinjection of tumor cells, YY1 mRNA levels in the xenografted tumors of mice after injection with SC-M1 cells transfected with YY1-expressing construct were higher than those of control cells (Supplementary Figure S6, *left*). The miR-34b (Supplementary Figure S6, *middle*) and miR-34c (Supplementary Figure S6, *right*) levels in the xenografted tumors of mice injected with miR-34bc-expressing SC-M1 cells were higher than those of control cells.

To investigate the role of endogenous miR-34 family-YY1 axis in metastatic colonization of gastric cancer cells, SC-M1 cells were co-transfected with antagomir-34a and siRNA vector against YY1 and then intravenously injected into lateral tail vein of NOD-SCID mice. Sixteen weeks later, mice were sacrificed for evaluation of metastatic nodules in lungs. Data showed that mice injected with YY1-knocked down SC-M1 cells had fewer and smaller lung metastatic nodules compared with those of control cells (Figure 7C). After transfection with antagomir-34a into SC-M1 cells, mice injected with the transfected cells had numerous large metastases in lung as compared with those injected with control cells. The augmented ability to form metastatic nodules in lungs by miR-34a knockdown was alleviated after YY1 knockdown.

The miRNA quantitative real-time PCR analysis was also performed on gastric cancer samples and the adjacent non-tumor tissues from 32 gastric cancer patients to examine their clinical relevance. Results showed that there were lower levels of miR-34a and miR-34c but not miR-34b in gastric tumor samples as compared with those in the corresponding adjacent normal tissues (Figure 7D).

## DISCUSSION

The multi-faceted protein YY1 [5-7, 25, 26] and miRNAs [11, 12] could function either oncogenic or tumor-suppressive roles in the tumorigenesis of hematopoietic malignancies and solid tumors. YY1 was found to cross-talk with miRNAs at least including miR-1 [20], miR-7 [19], miR-29 [15], miR-34a [10, 17, 18], miR-190 [13, 14], and miR-206 [16]. It was speculated that this combinatorial control may more precisely tune the levels of their down-stream target genes as compared with regulation by either mechanism alone [20]. The miR-34a had been shown to target YY1 [10, 17, 18] and the miR-34a-YY1 pathway was demonstrated to regulate expression of epidermal growth factor receptor in glioblastoma multiforme [18]. The biological function of miR-34a-YY1 pathway is still faint, especially its *in vivo* role. We demonstrate herein that miR-34 family-YY1 axis is involved in the development and progression of gastric cancer cells *in vitro* and *in vivo*.

In transcriptional control, YY1 plays a role in inducing chromatin remodeling besides acting as a repressor, activator, and initiator [6]. For example, the NF-kB-YY1 pathway suppresses miR-29 epigenetically and the regulatory circuitry of NF-kB-YY1-miR-29 is involved in skeletal myogenesis and rhabdomyosarcoma [15]. Further studies are needed to unravel whether the epigenetic status in host genes of miR-34 family is regulated by YY1 and the YY1-dependent epigenetic control of miR-34 family participates in regulating gastric carcinogenesis. In addition to translational regulation by miRNAs, it was suggested that the 3'-UTR of mRNA may attract cellular miRNAs and subsequently attenuates miRNA activities through interaction with them [27]. Possibly, the 3'-UTR of YY1 is involved in the modulating gene expression and regulating biological function *via* being targeted by miRNAs.

The identified targets of miR-34a include Bcl-2, cyclin D1, cyclin E2, CDK4, CDK6, E2F3, Met, SIRT1, Notch1, DLL1, CD44, AXIN2, and Pdgfrβ [28-31]. The miR-34b or miR-34c directly repress CDK6, CREB, E2F3, Met, c-Myc, CAV1, MYB, and SFRS2 [28]. It was demonstrated that the miR-34 family directly links tumor suppressor function and the oncogenic pathways in human cancer [32]. For example, p53-driven miR-34 family suppresses Wnt pathway and EMT via targeting the UTRs of Wnt- and EMT-related genes such as WNT1, WNT3, LRP6, AXIN2, β-catenin, LEF1 and Snail [30, 32, 33]. Besides, Arf-mediated miR-34a targets 3'-UTR of Pdgfrβ through a p53-independent manner [31]. All members of miR-34 family target 3'-UTR of YY1 mRNA (Figure 2D) and down-regulate the expression of YY1 in gastric cancer cells (Figure 2B). Although results of expression analysis for the affected mRNAs were nearly indistinguishable after individual transfection of miR-34 family, the binding affinities for c-Myc 3'-UTR among miR-34 family members could be different [28, 34]. Which of the miR-34 family members could exert the main effect on gastric carcinogenesis? Based on the results of Figure 7D, expressions of miR-34a in gastric tumor samples and the corresponding adjacent normal tissues were higher than those of miR-34c and miR-34b. Levels of miR-34a and miR-34c but not miR-34b in gastric tumor samples were lower than those in normal tissues (Figure 7D). In addition to the difference in binding affinities for their targets, the correlation between the expression levels and functions of miR-34a, miR-34b, and miR-34c in gastric cancer is still unclear so far.

In mice, the ubiquitous miR-34a is most highly expressed in brain, whereas miR-34b and miR-34c are present at highest levels in lung [35]. The miR-34a expression is higher than miR-34b and miR-34c levels in tissues of mice besides lung [28, 35]. In human gastric cancer cells, miR-34a is abundantly expressed in MKN74 and AGS cells, but miR-34b and miR-34c are epigenetically silenced by hypermethylation of CpG island in several gastric cancer cells [24]. Based on the results of Figure 3A, miR-34a is abundantly expressed in AZ521, AGS, NCI-N87, and SC-M1 gastric cancer cells but not in KATO III, NUGC-3, and SNU-16 cells.

It is suggested that YY1 [6, 36] and miRNAs [11, 12] have the potential as prognostic markers and therapeutic targets of tumors. A seven-miRNA signature was identified and associated with overall and relapse-free survival of gastric cancer patients [37]. The miRNAs from plasma and tumor tissues of patients could be potentially diagnostic and prognostic markers of gastric cancer [12, 38, 39]. Besides surgical resection, the signature of prognostic markers would be used to consider further adjuvant treatment for patients of gastric cancer [37]. Levels of miRNAs were effectively attenuated by antagomirs and unconjugated locked-nucleic-acid-modified oligonucleotides in animals [40, 41]. In 2013, a miR-34 mimic (MRX34) had been tested in phase 1 clinical trials in patients [29]. Possibly, the miRNA-based therapy could either restore miRNAs acting as tumor suppressors or down-regulate those acting as oncogenes for the treatment of cancers in the future [11]. However, the miRNA expression patterns in plasma and tumor samples might have some heterogeneity depending on sample types and cancer stages [12]. It is still a scientific and clinical challenge to overcome the complex and wide regulation of miRNA-mediated gene expression.

## MATERIALS AND METHODS

### Ethics Statement

Investigation has been conducted in accordance with the ethical standards and according to the Declaration of Helsinki and according to national and international guidelines and has been approved by the authors' institutional review board.

### Plasmids and plasmid construction

The expression construct pCMV-YY1 contains the cDNA encoding the full-length human YY1 [42, 43]. For the knockdown of endogenous YY1, the target sequence was constructed in siRNA vector pLKO.1 as described [43]. The RNAi vector against luciferase, pLKO.1-shLuc, was used as a negative control for knockdown validation. As described previously [44], the DNA segments of full-length (nucleotide 1-867 from the start of 3'-UTR) and truncated (nucleotide 449 to 867) YY1 3'-UTRs were amplified by PCR to construct reporter plasmids pYY1-3'-UTR-Luc (FL) and pYY1-3'-UTR-Luc (449-867), respectively. The luciferase reporter plasmids Nanog-Luc, SOX-2-Luc, and Oct4-Luc contain promoters of human pluripotency genes Nanog, SOX-2, and Oct4, respectively [45]. For the construction of adenoviral plasmids expressing miR-34a and miR-34b as well as miR-34c, their precursor sequences were amplified by PCR from the genomic DNA of SC-M1 cells. The precursor DNA fragments were cloned into shuttle vector pAdTrack-CMV (Stratagene) with a GFP tracer as described [44]. The primers used in plasmid construction are listed in the Supplementary Tables S1. All constructs were verified by sequencing.

### Cell culture and transfection

Human stomach adenocarcinoma SC-M1, AGS, AZ521, NUGC-3, KATO III, NCI-N87, and SNU-16 cells were cultured in RPMI 1640 medium with 10% fetal bovine serum. Cells were transiently transfected by electroporation or Lipofectamine™ 2000 (Invitrogen) transfection reagent. For luciferase reporter gene assay, SC-M1 cells (5 × 10^5^) were seeded onto 6-well plates and then transiently transfected [44]. Twenty-four hours after transfection, the transfected cells were infected with adenoviruses expressing miRNAs or GFP. Twenty-four hours after infection, luciferase activity was measured and then normalized [44]. Antagomir-34a, antagomir-34b, antagomir-34c, and scrambled control oligonucleotides (Ambion) were transfected into cells using the Lipofectamine™ 2000 (Invitrogen) according to the manufacturer's instructions.

### Recombinant adenoviruses

As described before [44], the recombinant adenoviral plasmids expressing miR-34a and miR-34b as well as miR-34c were constructed and then used to generate packaged recombinant adenoviruses expressing miR-34a (designated Ad-miR-34a) and miR-34b as well as miR-34c (designated Ad-miR-34bc). The vector pAdTrack-CMV was also used to obtain recombinant adenoviruses expressing GFP as a control (designated Ad-GFP).

### Quantitative real-time PCR analysis

For the detection of mRNAs, total RNA extracted by Trizol reagent (Invitrogen) was used to synthesize cDNA, then the cDNAs were amplified as described previously [46]. The primers used in real-time PCR analysis are listed in the Supplementary Tables S1. The relative quantification of mRNA level was normalized to that of GAPDH and corrected to a calibrator using the StepOne software 2.1 (Applied Biosystems). For the detection of mature miRNAs, cDNA synthesis and TaqMan miRNA real-time PCR assays were performed as described before [44]. Then the relative quantification of miRNA level was normalized with the level of RNU48 small nucleolar RNA and corrected to a calibrator using the StepOne software 2.1.

### Western blot analysis

Whole-cell extracts were prepared as described previously [47]. Western blotting was performed with anti-E-cadherin, anti-YY1, anti-plakoglobin, anti-vimentin (Santa Cruz), anti-N-cadherin (BD Biosciences), anti-CD44 (GeneTex), anti-Nanog (GeneTex), anti-SOX-2 (GeneTex), anti-Oct4 (GeneTex), and anti-GAPDH antibodies (Biogenesis).

### Flow cytometry analysis

Cells were washed with ice-cold phosphate-buffered saline (PBS) and fixed with slow vortex in 70% ethanol at −20°C overnight. As described [43], PI was added and incubated on dark in room temperature for 30 min. Then, FACSCalibur flow cytometry (Becton-Dickinson) was used to measure the emitted fluorescence from the PI-DNA complex.

### Cell growth and viability assays

The transfected or infected cells were seeded and then counted by trypan blue exclusion method at the time indicated for the evaluation of cell growth. Additionally, cells were also seeded and subsequently assessed cell viability by MTT (Sigma-Aldrich) assay as described previously [48].

### Colony and tumorsphere formation assays

The transfected or infected cells were seeded for the assay of anchorage-independent growth as described previously [46]. Then the colonies larger than 0.1 mm in diameter were counted from 10 random fields under the microscope. A total of 150 or 200 transfected or infected cells were suspended in stem cell-selective conditions and then seeded onto 96-well ultra-low attachment plates (Corning) for the formation of tumorspheres [44].

### Migration and invasion assays

The transfected or infected cells were seeded onto 24-well plates and migration as well as invasion assays were performed as described previously [49]. The migrated or invaded cells were counted from 10 random fields under the microscope.

### Scanning electron microscopic analysis

For ultra-structural analysis, tumorspheres collected by sedimentation were washed with PBS twice and then fixed with 2% glutaraldehyde for 2 hours, followed by 1% osmium tetraoxide for another 2 hours. After washing by deionized water, samples were frozen at −80°C for at least 8 hours and subsequently dehydrated overnight by Freeze-Drying machine (VirTis Freezemobile 25ES). The lyophilized samples were subjected to sputter coating with gold nano particles for 120 seconds prior to the examination with a scanning electron microscope (JSM-7600F; JEOL Ltd., Japan) at an accelerating voltage of 5 kV.

### Xenografted tumorigenicity assay in nude mice

All animal experiment protocols were carried out in accordance with a protocol approved by the institutional ethical committee in this study. Mice (BALB/c nu/nu) aged 5 weeks were subcutaneously injected with cells and tumor volume was estimated every 3 days [47, 49]. On day 30, the mice were sacrificed and then the expressions of mature miRNAs in excised tumor samples were detected by miRNA quantitative real-time PCR.

### *In vivo* tail vein metastasis assay

By tail vein injection [47], cells were injected into female non-obese diabetic severe-combined immunodeficiency (NOD-SCID) mice (National Taiwan University, Taipei, Taiwan) aged 5 weeks. The injected mice were sacrificed 16 weeks later and the metastatic nodules in lungs of mice were counted by gross and microscopic examination.

### Surgical samples

Tissues of human gastric adenocarcinoma were collected from gastric cancer patients who underwent gastric resection at the Department of Surgery, Taipei Veterans General Hospital. None of these patients had undergone chemotherapy or radiotherapy before surgery. Informed consent was obtained from all patients before study. The analysis of human tissue specimen was approved by the Institutional Review Board in Taipei Veterans General Hospital.

### Statistical analysis

Statistical analysis was performed using Student's t-test for simple comparison of two groups. The association between miR-34 family level analyzed by miRNA quantitative real-time PCR analysis and YY1 expression detected by Western blot analysis and subsequently quantified by Multi-Gouge V3.0 was analyzed using the Pearson correlation analysis. The difference was considered statistically significant when the P value was less than 0.05.

## SUPPLEMENTARY MATERIAL, TABLE AND FIGURES



## Abbreviations:

YY1: Yin Yang 1
miR-34: microRNA-34
EMT: epithelial-mesenchymal transition
MTT: 3-(4,5-dimethyl-2-thiazolyl)-2,5-diphenyl tetrazolium bromide; 3'-UTR,3'-untranslated region
TCGA: The Cancer Genome Atlas
