# Supplementary material for: Yin Yang 1 is a target of microRNA-34 family and contributes to gastric carcinogenesis
Source: Oncotarget. 2014 Jun 7;5(13):5002–16. doi: 10.18632/oncotarget.2073 (PMC4148117; doi:10.18632/oncotarget.2073)
Supplement: Supplementary file 1 [file oncotarget-05-5002-s001.pdf]

Yin Yang 1 is a target of microRNA-34 family and contributes to gastric carcinogenesis

Supplementary Material

Table S1: Sequence of primers for siRNA, PCR, and real-time PCR

Assays			Sequence (5′ to 3′)	Amplicon (bp)
siRNA	YY1		CCTCCTGATTATTCAGAATAT	
PCR	YY1 3′-UTR (full-length)	F	GCTCTAGAAAGAAGAGAGAAGACCC	883
		R	GCTCTAGACTTTAGGATTGCTATTTTATT	
	YY1 3′-UTR (449-867)	F	GCTCTAGAGATATGCTTAGTAATGCTA	465
		R	GCTCTAGACTTTAGGATTGCTATTTTATT	
	miR-34a	F	ATACTCGAGTGTGCCTTTTTCCTTC	381
		R	ATAAGATCTTCCTGCATCCTTTCTT	
	miR-34bc	F	ATAAGATCTAGCCATGGTAGGGCGT	786
		R	ATACTCGAGCAATTAATAGGCAAT	
real-time PCR	CD44	F	TCCAACACCTCCCAGTATGACA	83
		R	GGCAGGTCTGTGACTGATGTACA	
	Nanog	F	CCTGTGATTTGTGGGCCTG	78
		R	GACAGTCTCCGTGTGAGGCAT	
	Oct4	F	GGTGGAGGAAGCTGACAACAA	123
		R	AAATTCTCCAGGTTGCCTCTCA	
	SOX-2	F	GTATCAGGAGTTGTCAAGGCAGAG	78
		R	TCCTAGTCTTAAAGAGGCAGCAAAC	
	YY1	F	CCCACGGTCCCAGAGTCCA	162
		R	GTGTGCGCAAATTGAAGTCCAGT	
	GAPDH	F	AAATCCCATCACCATCTTCC	194
		R	TCACACCCATGACGAACA	

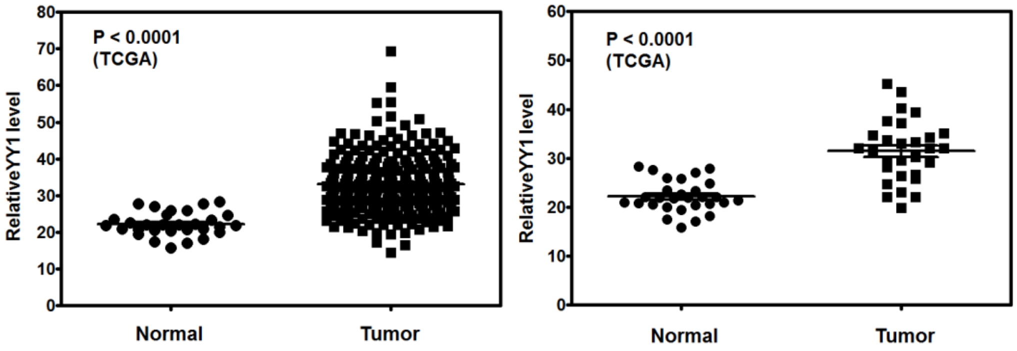

Figure S1: Levels of YY1 mRNA are significantly up-regulated in stomach adenocarcinoma specimens. Level_3 RNA-Seq data (Illumina Hiseq) from stomach adenocarcinoma samples and normal tissue samples were downloaded from the TCGA and Broad GDAC Firehose data portal. The RPKM (Reads per Kilobase of exon model per Million) values of all samples were selected and analyzed for comparing abundances by GraphPad Prism 5 software. The transcript levels of YY1 in stomach adenocarcinoma samples (n= 241) and normal tissue samples (n= 30) were measured by RNA sequencing in TCGA data (*left*). Analysis using TCGA data was also performed to compare the levels of YY1 mRNA between stomach adenocarcinoma and their corresponding normal-tissue counterparts from 30 patients with stomach adenocarcinoma (*right*).

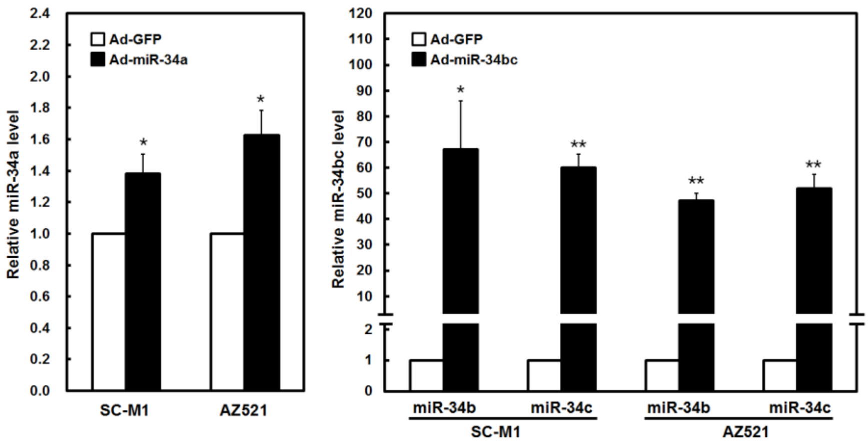

Figure S2: Establishment of miR-34 family-expressing adenoviral system in SC-M1 and AZ521 gastric cancer cells. SC-M1 and AZ521 cells were infected with adenoviruses expressing miR-34a (Ad-miR-34a), miR-34b and miR-34c (Ad-miR-34bc), or GFP (Ad-GFP) for 48 hours. The relative levels of miR-34a (*left*) and miR-34b as well as miR-34c (*right*) were determined using miRNA quantitative real-time PCR in the infected SC-M1 and AZ521 cells. The levels of miR-34 family in SC-M1 or AZ521 cells were set to unity. *, $P < 0.05$; **, $P < 0.01$ compared with cells infected with GFP-expressing adenoviruses.

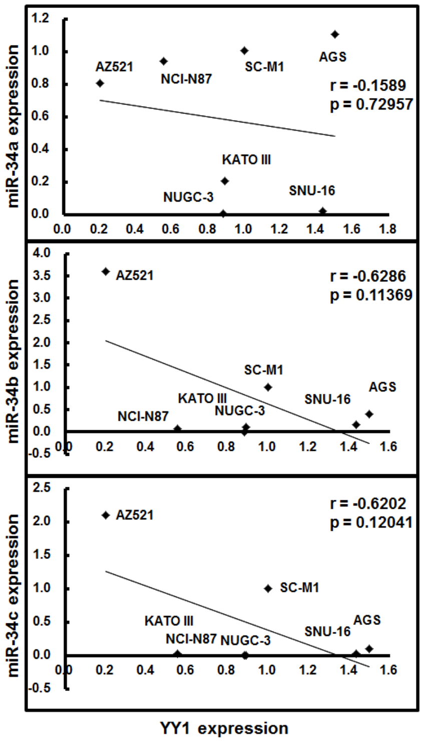

Figure S3: Correlation between YY1 and miR-34 family levels in gastric cancer cells. The correlation between miR-34 family (Figure 3A) and YY1 protein (Figure 3B) expressions in seven gastric cancer cells was analyzed by Pearson correlation analysis as described in the legend to Figure 3.

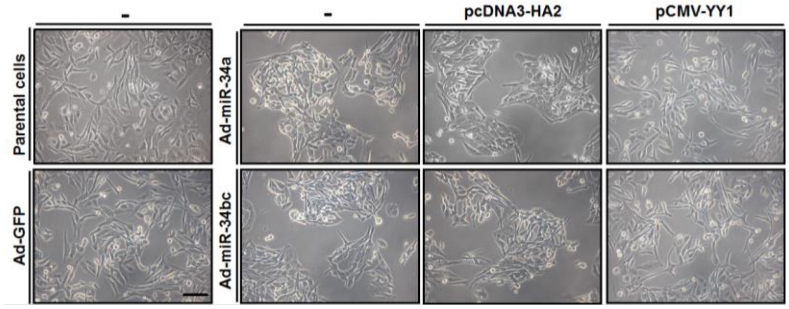

Figure S4: The miR-34 family induces morphological change of NUGC-3 gastric cancer cells through targeting YY1. NUGC-3 cells were transfected with YY1-expressing construct pCMV-YY1 or control vector pcDNA3-HA2 and subsequently infected with adenoviruses expressing miR-34a (Ad-miR-34a), miR-34b and miR-34c (Ad-miR-34bc), or GFP (Ad-GFP) as described in the legend to Figure 4A. The treated cells were seeded onto 6-well plates for 48 hours for morphological examination. -, cells without transfection. Bar, 100 μm.

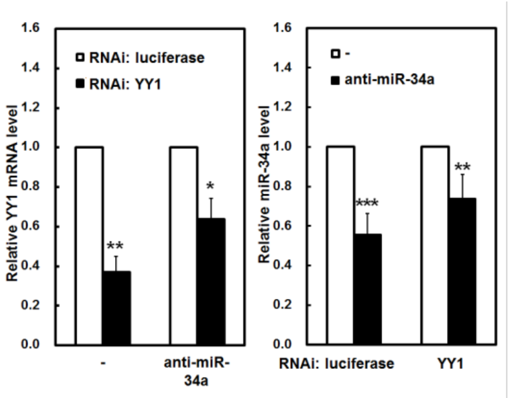

Figure S5: The knockdown of YY1 and miR-34a in the xenografted tumors of mice after injection with SC-M1 cells transfected with antagomir-34a and siRNA vector against YY1. As described in the legend to Figure 7A, the SC-M1 cells co-transfected with antogomir-34a and siRNA vector against YY1 were subcutaneously injected into nude mice (n= 5 per group). On day 30, the mice were sacrificed and subcutaneous tumors were excised for the detection of YY1 mRNA (*left*) and miR-34a (*right*) expressions using quantitative real-time PCR. *, P < 0.05; **, P < 0.01; ***, P < 0.001 compared with cells transfected with siRNA vector against luciferase for the detection of YY1 mRNA or transfected with scrambled control for the detection of miR-34a.

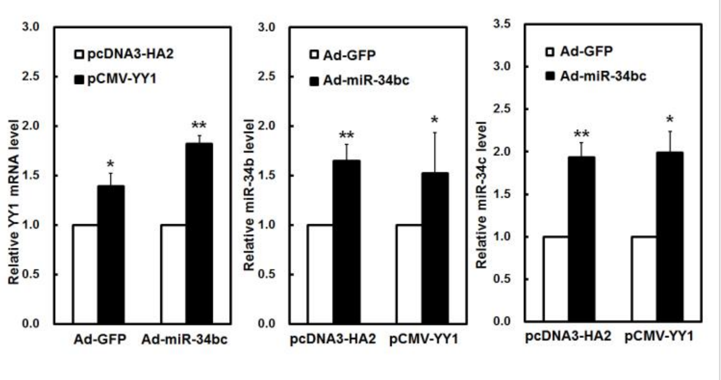

Figure S6: The exogenous expressions of YY1 and miR-34b as well as miR-34c in the xenografted tumors of mice after injection with SC-M1 cells transfected with YY1-expressing construct and infected with adenoviruses expressing miR-34b and miR-34c. As described in the legend to Figure 7B, the SC-M1 cells transfected with antogomir-34a and subsequently infected with adenoviruses expressing miR-34b and miR-34c (Ad-miR-34bc) were subcutaneously injected into nude mice (n= 5 per group). On day 30, the mice were sacrificed and subcutaneous tumors were excised for the detection of YY1 mRNA (*left*), miR-34b (*middle*), and miR-34c (*right*) expressions using quantitative real-time PCR. *, $P < 0.05$; **, $P < 0.01$ compared with cells transfected with control vector pcDNA3-HA2 for the detection of YY1 mRNA or infected with adenoviruses expressing GFP for the detection of miR-34b and miR-34c.
